# Pneumococcal Meningovasculitis: A Deadly Foe

**DOI:** 10.7759/cureus.80954

**Published:** 2025-03-21

**Authors:** Filipa Côrte-Real, Catarina Lume, Hugo Dória, Ana Marta Mota, José J Nóbrega

**Affiliations:** 1 Intensive Care Department, Hospital Central do Funchal, Funchal, PRT; 2 Radiology Department, Hospital Central do Funchal, Funchal, PRT

**Keywords:** encephalopathy, fulminant infection, meningovascular involvement, neurocritical patient, pneumococcal meningitis

## Abstract

Meningitis is an inflammation of the protective membranes surrounding the brain and spinal cord, typically caused by viral or bacterial infections. Bacterial meningitis is a medical emergency requiring prompt diagnosis and treatment, which is associated with a significant mortality rate, even with optimal care.

We report the case of a previously healthy 57-year-old man who presented to the emergency department with a three-week history of fever, dry cough, myalgia, fatigue, weakness, anorexia, and excessive sweating. Physical examination revealed a tympanic temperature of 38.9ºC, leukocytosis with neutrophilia, and elevated C-reactive protein levels. A computed tomography (CT) scan of the chest, abdomen, and pelvis showed consolidations in the left lung with air bronchograms and ground-glass opacities. His condition rapidly deteriorated with progressively higher fever, neck stiffness, positive Kernig and Brudzinski signs, profuse sweating, and altered mental status with a Glasgow Coma Scale (GCS) of 8, requiring non-invasive ventilation. He was thus admitted to the intensive care unit (ICU) on the second day of hospitalization. Lumbar puncture confirmed Streptococcus pneumoniae in the cerebrospinal fluid culture, supporting the diagnosis of pneumococcal meningitis. Electroencephalogram (EEG) findings were consistent with severe encephalopathy, while a follow-up CT scan revealed bilateral temporal hypodensities extending into the brainstem, suggestive of ischemic lesions of a vasculitic nature. Magnetic resonance imaging (MRI) confirmed multiple acute ischemic lesions throughout the brain and brainstem, along with signs of leptomeningitis and purulent collections in the lateral ventricles. Despite receiving 14 days of antibiotic therapy and intensive medical efforts, the patient showed no clinical improvement and ultimately succumbed to the infection. This case highlights the critical importance of early recognition and diagnosis of meningitis, as delayed treatment can lead to devastating outcomes. Continuous monitoring and a low threshold of suspicion are key elements for preventing fulminant central nervous system infections.

## Introduction

Meningitis is a severe inflammatory condition affecting the protective membranes surrounding the brain and spinal cord. It can be caused by viral, bacterial, fungal, or parasitic infections, with bacterial meningitis being the most life-threatening form. Despite advances in medical care, bacterial meningitis remains a significant global health concern due to its significant mortality and morbidity rates [1,2,3]. Bacterial meningitis has an annual incidence of approximately 0.9 cases per 100,000 adults in developed countries, with higher rates in low-income regions [2]. Pneumococcal meningitis is a severe and potentially fatal infection, often presenting with nonspecific initial symptoms that can delay diagnosis and treatment [2].

We report the case of a 57-year-old male who initially exhibited only mild systemic symptoms, with fever as the most prominent feature. His condition rapidly deteriorated, requiring intensive care support, and he was later found to have aggressive vascular involvement. This case highlights the unpredictable and fulminant nature of pneumococcal meningitis and underscores the critical importance of early diagnosis and timely intervention. Prompt recognition and treatment are crucial to improving patient outcomes, yet the disease often presents with nonspecific symptoms, leading to delayed diagnosis. Even a seemingly mild presentation can rapidly progress into a life-threatening condition with devastating complications.

## Case presentation

**Figure 1:**
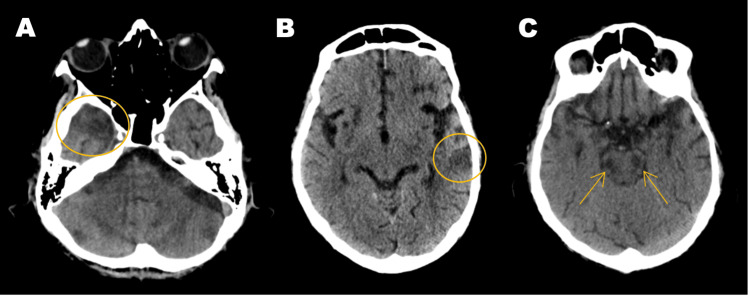
Brain CT scan A-C: In the follow-up CT scan, new areas of hypodensity were seen in the temporal lobes bilaterally (circles in A and B) and in the brainstem (arrows in C).

**Figure 2:**
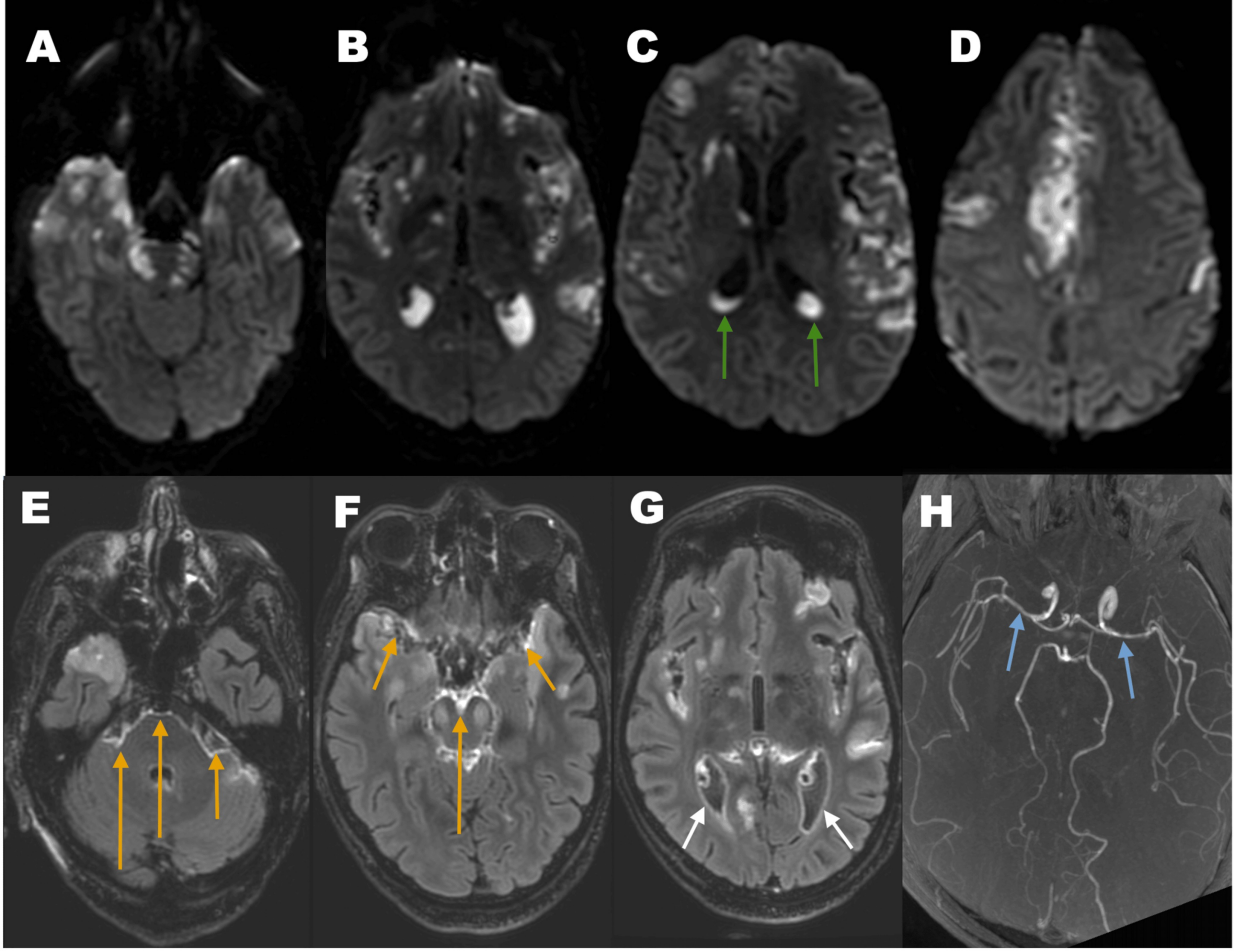
Brain MRI scan A-D: diffusion-weighted images (DWI) on the axial plane showing multiple bright areas scattered throughout the fronto-temporal lobes and on the pons bilaterally that are compatible with acute infarcts, as well as pus inside the ventricles (green arrows); E-G: post-contrast T2-FLAIR images on the axial plane, besides the parenchymal lesions, there’s also leptomeningeal (orange arrows) and ependymal (white arrows) enhancement in keeping with meningitis and ventriculitis, respectively; H: 3D time-of-flight (TOF) angiography with areas of vasoconstriction on both carotid tips and middle cerebral arteries, suggestive of vasculitis (blue arrows). FLAIR: fluid attenuated inversion recovery

A 57-year-old male, a taxi driver with a 60-pack-year smoking history and no known comorbidities or regular medication, presented to the emergency department with a three-week history of fever, dry cough, fatigue, weakness, anorexia, and excessive sweating. On admission, his physical examination was unremarkable except for a tympanic temperature of 38.9ºC (Table 1). Initial laboratory findings revealed leukocytosis (20,000/μL) with neutrophilia (91%), an elevated C-reactive protein (CRP) level of 111 mg/L, and thrombocytopenia (93,000/μL). Arterial blood gas analysis (on room air) showed a pH of 7.52, partial pressure of carbon dioxide (CO₂) of 29.1 mmHg, partial pressure of oxygen (pO₂) of 82.4 mmHg, bicarbonate (HCO₃) of 25.9 mEq/L, potassium of 3.2 mEq/L, sodium of 135 mEq/L, chloride of 101 mEq/L, lactate of 1.0 mmol/L, and glucose of 156 mg/dL (Table 2). COVID-19 and influenza tests were negative. On the first day of hospitalization in the internal medicine ward, the patient remained febrile without an apparent infectious focus. A chest, abdominal, and pelvic CT scan was performed to investigate the persistent fever, revealing consolidations in the lower and upper lobes of the left lung with air bronchograms and ground-glass opacities, findings suggestive of pneumonia. Empirical antibiotic therapy with amoxicillin-clavulanic acid and clarithromycin was initiated. On the second day, there was a sudden deterioration in mental status and respiratory distress, leading to the activation of the hospital’s emergency response team. He was noted to have a Glasgow Coma Scale (GCS) score of 8, neck stiffness, positive Kernig and Brudzinski signs, and profuse sweating. His respiratory rate increased to 53 breaths per minute, blood pressure was 130/80 mmHg, and heart rate was 114 bpm (Table 1). Arterial blood gas analysis (on room air) showed a pH of 7.43, pCO₂ of 37 mmHg, pO₂ of 87 mmHg, HCO₃ of 25 mEq/L, oxygen saturation of 96.8%, and lactate of 1.9 mmol/L (Table 2). Given his worsening neurological and respiratory status, the patient was intubated, started invasive mechanical ventilation, and transferred to the intensive care unit (ICU). A lumbar puncture was performed, revealing cerebrospinal fluid (CSF) findings consistent with bacterial meningitis: 7,960 cells/μL, predominantly polymorphonuclear leukocytes, glucose <2 mg/dL, and protein >600 mg/dL. Empirical antibiotic therapy was subsequently changed to ampicillin, ceftriaxone, and vancomycin. On the third ICU day, CSF culture confirmed Streptococcus pneumoniae as the causative pathogen. Peripheral blood cultures also grew Streptococcus pneumoniae, while sputum cultures were negative. Based on these results, ampicillin and vancomycin were discontinued, and dexamethasone was introduced to manage CNS inflammation. By the fifth ICU day, the patient exhibited pathological awakening, with fluctuating GCS scores between 6 and 8. An electroencephalogram (EEG) showed diffuse severe encephalopathy characterized by delta slowing. A CT brain scan revealed extensive bilateral temporal hypodensities extending into the brainstem, more pronounced on the right, raising the suspicion of vasculitic lesions (Figure 1), prompting further evaluation with MRI. On the seventh ICU day, MRI findings (Figure 2) confirmed the presence of meningoencephalitis with multiple acute ischemic lesions throughout both cerebral hemispheres and the brainstem. Additionally, purulent collections were detected within the ventricles, and there was an ependymal enhancement in keeping with ventriculitis. The imaging findings were consistent with a fulminant course of pneumococcal meningitis complicated with vasculitis. Despite 14 days of appropriate antibiotic therapy, the patient showed no signs of clinical improvement. His neurological status remained severely compromised, and he ultimately succumbed to the infection despite intensive medical efforts. Upon reviewing the clinical records, it is important to note that the patient had not received pneumococcal vaccination. This highlights the crucial role of preventive strategies in reducing the burden of bacterial meningitis.

**Table 1:** Vital signs from the patient

Vital signs	Day of admission	Two days after admission
Respiratory rate	18 bpm	53 bpm
Peripheral oxygen saturation	94%	95%
Heart rate	87 bpm	114 bpm
Blood pressure	142/88 mmHg	130/80 mmHg
Glasgow Coma Scale	15 (Eyes 4; Verbal 5; Motor 6)	8 (Eyes 2; Verbal 1; Motor 5)
Tympanic temperature	38.9ºC	38.7ºC

**Table 2:** Laboratory data from the patient pCO₂: partial pressure of carbon dioxide; pO₂: partial pressure of oxygen; HCO₃: bicarbonate

Parameter	Day of admission	Two days after admission	Reference range
Leukocytes	20,000/μL	21,800/μL	4,200-10,800/μL
Neutrophils	91%	92.7%	-
C-reactive protein (CRP)	111 mg/L	100.6 mg/L	< 5 mg/L
Platelets	93,000/μL	190,000//μL	144,000-440,000/μL
pH	7.52	7.43	7.35-7.45
pCO₂	29.1 mmHg	37 mmHg	35-45 mmHg
pO₂	82.4 mmHg	87 mmHg	80-100 mmHg
HCO₃	25.9 mEq/L	25 mEq/L	21-26 mEq/L
Potassium	3.2 mEq/L	4.0 mEq/L	3.5-5.1 mEq/L
Sodium	135 mEq/L	140 mEq/L	134-145 mEq/L
Chloride	101 mEq/L	103 mEq/L	95-105 mEq/L
Lactate	1.0 mmol/L	1.9 mmol/L	0.4-2 mmol/L
Glucose	156 mg/dL	180 mg/dL	74-106 mg/dL
COVID-19 test	Negative	-	-
Influenza test	Negative	-	-
Cerebrospinal fluid			
Cells	-	7,960 cells/μL	<5 cells/μL
Predominance	-	Polymorphonuclear	Lymphomononuclear
Glucose	-	<2 mg/dL	50-80 mg/dL
Protein	-	>600 mg/dL	15-45 mg/dL

## Discussion

Streptococcus pneumoniae and Neisseria meningitidis are the primary bacterial agents responsible for community-acquired meningitis in adults [2]. Despite significant progress in antibiotic treatments and critical care management, bacterial meningitis remains a life-threatening condition, especially when complications such as meningovascular involvement arise.

Pneumococcal meningitis is associated with a particularly poor prognosis, with mortality rates of up to 54% in low-income countries and a high likelihood of long-term neurological sequelae among survivors [2,4]. The classic triad of bacterial meningitis includes headache (84%), fever (74%), and neck stiffness (74%), though not all patients present with these symptoms simultaneously [2]. Other common manifestations include altered mental status, photophobia, nausea, vomiting, and focal neurological deficits. Severe cases, as seen in our patient, may rapidly progress to coma and require intensive care. The presence of Kernig and Brudzinski signs, though not highly sensitive, is strongly suggestive of meningeal irritation [2]. Laboratory findings typically reveal leukocytosis, neutrophilia, and elevated inflammatory markers, while cerebrospinal fluid (CSF) analysis remains the gold standard for diagnosis, showing pleocytosis, elevated protein, and decreased glucose levels.

Vascular injury in bacterial meningitis occurs due to a combination of inflammatory processes, endothelial activation, and thrombosis, leading to ischemia and dysfunction of the blood-brain barrier (BBB) [5,6]. Infection by pathogens (e.g., Streptococcus pneumoniae, Neisseria meningitidis, Haemophilus influenzae) activates the innate immune response in the subarachnoid space. Macrophages and microglial cells release pro-inflammatory cytokines (TNF-α, IL-1β, IL-6), increasing vascular permeability and attracting neutrophils. Inflammation causes dysfunction of astrocytes and pericytes, leading to the loss of BBB integrity [6]. The damaged endothelium expresses adhesion molecules (ICAM-1, VCAM-1), promoting further leukocyte recruitment. Consequently, vascular permeability increases, resulting in cerebral edema and elevated intracranial pressure [6]. Simultaneously, inflammation activates the coagulation cascade, leading to the formation of microthrombi in cerebral vessels, contributing to ischemia and cerebral infarcts [6]. Additionally, inflammatory cytokines induce inflammation of meningeal vessels, leading to vasculitis and impaired blood flow. Vascular spasms may also occur, further reducing cerebral perfusion and increasing the risk of ischemic stroke [6].

Meningovasculitis is a severe complication of bacterial leptomeningitis, characterized by inflammation-induced vascular damage, leading to ischemic or hemorrhagic strokes [5]. This occurs due to direct bacterial invasion, cytokine-mediated endothelial dysfunction, and/or immune-mediated vasculitis. In pneumococcal meningitis, vasculitic changes can affect large and small vessels, particularly in the basal regions of the brain [5]. Imaging studies, such as MRI, often reveal ischemic lesions, particularly in the cortex, basal ganglia, and brainstem, as observed in our patient. The presence of bilateral temporal hypodensities extending into the midbrain strongly suggested a vasculitic process secondary to pneumococcal meningitis [5].

Early and aggressive treatment is crucial in bacterial meningitis to improve outcomes. Empirical antibiotic therapy should be initiated immediately upon clinical suspicion, typically with a combination of a third-generation cephalosporin (e.g., ceftriaxone or cefotaxime), vancomycin to cover resistant strains, and adjunctive dexamethasone to reduce inflammation and neurological complications [2,3,4,5]. Once S. pneumoniae is confirmed, therapy can be tailored accordingly, often continuing high-dose intravenous penicillin or ceftriaxone for 10-14 days [1,4]. In cases complicated with vasculitis, additional supportive measures such as anticoagulation and corticosteroids have been considered, although their role remains controversial. Continuous monitoring in an intensive care setting is essential, particularly in patients with altered mental status or respiratory compromise. Despite these interventions, mortality remains high, as demonstrated in this case, emphasizing the aggressive nature of pneumococcal meningitis with vascular involvement. 

Survivors of bacterial meningitis often experience long-term complications, including cognitive impairment, hearing loss, seizures, and motor deficits [3]. Therefore, post-recovery follow-up should include neurological and neuropsychological assessments, as well as hearing evaluations. In cases with meningovascular involvement, prolonged rehabilitation may be necessary due to stroke-related disabilities. Preventive measures, including pneumococcal and meningococcal vaccination, remain the most effective strategy to reduce the incidence of bacterial meningitis [3,4].

## Conclusions

Despite optimal medical care, bacterial meningitis remains a highly lethal condition, reinforcing the need for preventive strategies such as vaccination and early intervention in suspected cases. Although appropriate antibiotic therapy and intensive medical care were provided, the patient’s condition rapidly deteriorated, ultimately leading to a fatal outcome. The case underscores the importance of continuous monitoring, early neurological assessment, and aggressive management strategies to prevent severe complications. Increased awareness, a low threshold of suspicion, and timely intervention remain essential to improve prognosis and reduce the burden of bacterial meningitis.
